# Short-term amino acid infusion improves protein balance in critically ill patients

**DOI:** 10.1186/s13054-015-0844-6

**Published:** 2015-03-12

**Authors:** Felix Liebau, Martin Sundström, Luc JC van Loon, Jan Wernerman, Olav Rooyackers

**Affiliations:** Division of Anesthesia and Intensive Care, Department of Clinical Sciences, Intervention and Technology (CLINTEC), Karolinska Institutet and Karolinska University Hospital, Hälsovägen 13, Huddinge, 14186 Sweden; NUTRIM School for Nutrition, Toxicology and Metabolism, Maastricht University, Universiteitssingel 40, Maastricht, 6229ER The Netherlands

## Abstract

**Introduction:**

Evidence behind the recommendations for protein feeding during critical illness is weak. Mechanistic studies are needed to elucidate the effects of amino acid and/or protein supplementation on protein metabolism before larger clinical trials with higher levels of protein feeding are initiated.

**Methods:**

We studied the effects of parenteral amino acid supplementation (equivalent to 1 g/kg/day) over the course of 3 hours on whole-body protein turnover in critically ill patients in the intensive care unit (ICU) during the first week after admission. Patients were studied at baseline during ongoing nutrition and during extra amino acid supplementation. If the patient was still in the ICU 2 to 4 days later, these measurements were repeated. Protein kinetics were measured using continuous stable isotope-labeled phenylalanine and tyrosine infusions.

**Results:**

Thirteen patients were studied on the first study occasion only, and seven were studied twice. Parenteral amino acid supplementation significantly improved protein balance on both occasions, from a median of −4 to +7 μmol phenylalanine/kg/hr (*P* =0.001) on the first study day and from a median of 0 to +12 μmol phenylalanine/kg/hr (*P* =0.018) on the second study day. The more positive protein balance was attributed to an increased protein synthesis rate, which reached statistical significance during the first measurement (from 58 to 65 μmol phenylalanine/kg/hr; n =13; *P* =0.007), but not during the second measurement (from 58 to 69 μmol phenylalanine/kg/hr; n =7; *P* =0.09). Amino acid oxidation rates, estimated by phenylalanine hydroxylation, did not increase during the 3-hour amino acid infusion. A positive correlation (*r* =0.80; *P* <0.0001) was observed between total amino acids and/or protein given to the patient and whole-body protein balance.

**Conclusion:**

Extra parenteral amino acids infused over a 3-hour period improved whole-body protein balance and did not increase amino acid oxidation rates in critically ill patients during the early phase (first week) of critical illness.

**Electronic supplementary material:**

The online version of this article (doi:10.1186/s13054-015-0844-6) contains supplementary material, which is available to authorized users.

## Introduction

Critically ill patients are characterized by a progressive loss of lean body mass, mainly confined to skeletal muscle mass. The loss of this lean body mass is related to a worsened outcome. The most obvious way to prevent this is by adequate nutrition, including protein feeding. Although the effects of varying caloric and protein supply have been addressed in clinical studies, the evidence underlying clinical recommendations for protein feeding in critically ill patients remains weak, as reviewed by Hoffer and Bistrian [1]. Current recommendations for protein intake in critically ill patients vary between 1.2 and 2.5 g/kg/day [1-3], also indicating the uncertainty of the scientific evidence for the recommendations. Smaller physiological, and subsequently larger, clinical trials are needed to solve this problem.

Protein requirements are mostly studied by measuring nitrogen balance in response to a certain diet. However, the validity of the nitrogen balance technique to assess the effects of different protein feeding regimens over a short period (less than a few weeks) has been questioned [4]. We have previously applied stable isotope amino acid tracer techniques to study the impact of varying feeding strategies on whole-body protein metabolism in critically ill patients. This methodology allows for a direct measurement of the impact of a feeding regimen on *in vivo* protein breakdown, protein synthesis, protein balance and amino acid oxidation. We found that patients with head trauma had a better protein balance when fed standard parenteral nutrition at 100% of measured energy expenditure (indirect calorimetry) than when fed 50% [5]. However, it remains unclear whether this effect is due to increases in the caloric supply, the amino acid supply, or both.

In the present study, we investigated the effect of an increased parenteral amino acid supply on top of ongoing nutrition in critically ill patients during the first week of intensive care unit (ICU) treatment. The first week was chosen because the largest deficit in cumulative nutritional supply is normally established in this patient group [6]. To achieve this, we first performed a pilot study to validate the use of a free stable isotope-labeled phenylalanine tracer to measure the splanchnic protein extraction during ongoing feeding. In the actual study, we then addressed three questions. The primary question was whether extra parenteral amino acids during the first week in the ICU modulate whole-body protein balance. The two secondary questions were whether baseline protein balance changes during early ICU treatment and whether the effects of extra parenteral amino acids are maintained after a few days.

## Materials and methods

### Pilot study

To measure whole-body protein turnover during ongoing enteral nutrition, two distinct tracers of the same amino acid need to be given, one parenterally and one enterally. This is necessary to correct the appearance of the enteral amino acids for the splanchnic extraction (that is, the fraction of dietary amino acid that is not available systemically). Because the digestion and absorption kinetics of protein may be different from those of free amino acids given enterally, the enteral tracer should preferentially be given within the matrix of the food provided [7,8]. Previous work has successfully produced intrinsically [1-^13^C]phenylalanine-labeled milk proteins by infusing Holstein cows with large amounts of tracer [7]. Although the kinetics of dietary intact protein versus free amino acids have been investigated using bolus feeding in healthy persons, the situation in critically ill patients may differ substantially because of the use of continuous feeding, physiological changes affecting gastric emptying, protein digestion and amino absorption, and the use of drugs such as opioids and proton pump inhibitors. We therefore first investigated whether the enteral tracer kinetics from an intrinsically labeled protein and a free amino acid might behave similarly after a few hours of continuous enteral feeding via a feeding tube. To test this hypothesis, we performed a pilot study in which six critically ill patients treated in the ICU received an enteral feed containing both intrinsically [1-^13^C]-phenylalanine-labeled casein [7] (isotopic enrichment 35.2% molar percent excess (MPE)) and a free [ring-^2^H_5_]phenylalanine tracer together with maltodextrin for 6 hours. None of the six patients had received any previous enteral nutrition while in the ICU. The enteral feed was given as 1.5 g/hr labeled casein (resulting in 0.068 g/hr phenylalanine), 2.7 g/hr maltodextrin and 0.068 g/hr free ^2^H_5_-phenylalanine. Plasma samples were taken from an existing arterial line and analyzed for both [^13^C]phenylalanine and [^2^H_5_]phenylalanine as described previously [9]. The rate of appearance estimated by the two enterally given tracers was calculated by dividing the infusion rate of each tracer given enterally by the plasma enrichment (in atom percent excess) per kilogram of body weight.

**Figure 1:**
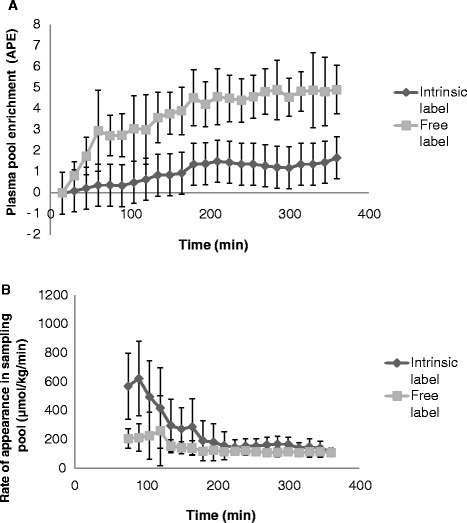
**Plasma enrichments (A) and rate of appearance (B) measured with** ^**13**^ **C-phenylalanine intrinsically labeled casein protein (35.2 molar percent excess (MPE)) and with the same amount of free [** ^**2**^ **H**_**5**_ **]phenylalanine (99.7 MPE) given enterally together with maltodextrin in six critically ill patients for 6 hours.** The rate of appearance is an apparent rate of appearance of phenylalanine (not corrected for splanchnic extraction) into the central circulation and is calculated by dividing the enteral infusion rate of the two tracers by the plasma enrichment of each tracer at each time point. In one patient, none of the tracers escaped the splanchnic tissues, so mean values and standard deviations of five patients are shown. After about 4 hours, the rate of appearance was identical for both tracers. The individual data for these patients are given in Additional file 1.

The patients’ characteristics are shown in Table 1. For one patient, neither of the two tracers was detected in arterial plasma. For the other five, the initial appearance was very different for the two tracers, with the free amino acid tracer appearing much faster. However, after 4 hours, the appearance rates were similar for the remaining 2 hours (see Figure 1 for mean values and Additional file 1 for individual curves). On the basis of these results, we conclude that whole-body protein kinetics measurements during ongoing continuous enteral nutrition in critically ill patients can be assessed by adding a free labeled phenylalanine tracer to the enteral nutrition and measuring after a 4-hour run-in period. This result also shows that the digestion capacity for casein is not compromised in these critically ill patients.

**Table 1:** **Patient characteristics**

	**Pilot study (n =6)**	**Q1 effect AA (n =13)**	**Q2 time effect basal (n =10, subgroup of Q1)**		**Q3 time effect AA (n =7, subgroup of Q2)**	
		**First study day**	**First study day**	**Second study day**	**First study day**	**Second study day**
Age, yr	70 (61 to 79)	69 (46 to 77)	71 (46 to 77)		71 (46 to 77)	
Sex, male/female	3/3	8/5	6/4		4/3	
Weight, kg	75 (63 to 95)	85 (42 to 146)	92 (70 to 146)		81 (70 to 138)	
Diagnosis, medical/surgical	2/4	7/6	4/6		3/4	
SAPS III	73 (41 to 76)	70 (54 to 93)	70 (54 to 93)		73 (54 to 93)	
Days in ICU	4 (3 to 48)	5 (3 to 7)	4 (3 to 6)	7 (5 to 10)*	4 (3 to 6)	7 (5 to 10)*
SOFA score on study day	8 (1 to 12)	7 (2 to 14)	6 (2 to 14)	7 (2 to 15)	7 (3 to 14)	5 (2 to 15)

Characteristics for the patients included in the pilot study and the different cohorts to evaluate the three research questions (Q1 through Q3). Q1: Do extra parenteral amino acids (AA) during the first week in the intensive care unit (ICU) modulate whole-body protein kinetics? Q2: Do baseline protein kinetics change during early ICU treatment? Q3: Are the effects of extra parenteral amino acids maintained after a few days? *Significantly different from first study day in same cohort (*P* <0.05 by Wilcoxon signed-rank test). SAPS III, Simplified Acute Physiology Score III; SOFA, Sequential Organ Failure Assessment.

### Parenteral amino acids study

In this study, we addressed three questions. The primary question was whether extra amino acids given as 3-hour parenteral supplementation can be used in critically ill patients to improve protein turnover and balance during the initial week after ICU admission. The two secondary questions were (1) whether basal protein turnover rates are stable during the initial phase of ICU stay and (2) whether the response to extra parenteral amino acids is maintained. We therefore recruited patients admitted to the general ICU at Karolinska University Hospital in Huddinge, Sweden, during the initial week after admission and studied them during ongoing parenteral and/or enteral nutrition. Patients were studied again if still present in the ICU 2 to 4 days later. Both this study and the pilot study were approved (2011/2019-31/1) by the regional ethical review board in Stockholm (etikprövningsnämnden). Patients and relatives were informed about the study orally and in writing before written informed consent was obtained.

#### Study protocol

The study was designed as a pragmatic intervention study, meaning that standard care, including nutritional support, was continued. Nutrition routines included early initiation of enteral nutrition and supplementary parenteral nutrition if needed after day 5 in the unit. Energy targets were set according to energy expenditure measured by indirect calorimetry or otherwise at 20 kcal/kg/day. Protein targets were set at 1.2 g/kg/day. Glutamine supplementation was given only to patients receiving parenteral nutrition who had low plasma glutamine concentrations (<500 μM). Nutrition was not changed during ongoing measurements for the study, except for any glutamine supplementation, which was stopped at least 8 hours before the start of the study. Whole-body protein kinetics were measured during the initial week of the ICU stay, first during the baseline situation and again following 3 hours of parenteral amino acid supplementation at an equivalent of 1 g/kg/day. A second, identical study was performed 2 to 4 days later if the patient was still in the ICU. Patients with ongoing hemodialysis, without arterial access for sampling, or younger than 18 years of age were excluded from participating in the study.

On the study day at time 0, ongoing enteral nutrition was supplemented with [1-^13^C]-phenylalanine to obtain a 30% enrichment (MPE) in the total phenylalanine content in the enteral nutrition. Two hours later (time (*t*) =2 hours) a primed continuous intravenous infusion of [ring-^2^H_5_]phenylalanine, [^2^H_4_-]tyrosine and [^2^H_2_]tyrosine was started. The prime consisted of 0.5 mg/kg [ring-^2^H_5_]phenylalanine, 0.15 mg/kg [ring-^2^H_4_]tyrosine and 0.3 mg/kg [3,3-^2^H_2_]tyrosine and was immediately followed by a continuous infusion of 0.5 mg/kg/hr [ring-^2^H_5_]phenylalanine and 0.3 mg/kg/hr [3,3-^2^H_2_]tyrosine. After another 3 hours (*t* =5 hours), an infusion with a mixture of amino acids (Glavamin; Fresenius Kabi, Uppsala, Sweden) was started in a central venous catheter at a rate of 0.083 g/kg/hr total amino acids (equivalent to 1 g/kg/day) and maintained for 3 hours (until *t* =8 hours). Ethylenediaminetetraacetic acid plasma samples were obtained just before time 0 and during the last half-hour of the baseline (*t* = 4.5 to 5 hours) and of the intervention period (*t* =7.5 to 8 hours). During each of the latter two periods, four samples were obtained at 10-minute intervals. Plasma samples were kept at −80°C until analysis by gas chromatography–mass spectrometry as described previously [9]. In addition, the last samples of the baseline and intervention periods were analyzed for amino acid concentrations [10] and urea (kit for Konelab; Thermo Scientific, Stockholm, Sweden). The calculated sum of total amino acids includes the amino acids given by the supplementation minus proline, which is not analyzed in our method.

#### Calculations and statistics

Whole-body protein kinetics, including protein breakdown (endogenous rate of appearance), phenylalanine oxidation (estimated by phenylalanine hydroxylation rate), protein synthesis (rate of disappearance minus phenylalanine hydroxylation rate), splanchnic extraction and protein balance (difference between breakdown and synthesis rates) were calculated using formulas published elsewhere [9,11]. Mean enrichments of the four plasma samples from each sampling period were used for these calculations.

All data are presented as median (range). The three questions addressed were statistically analyzed separately. Nonparametric analyses were performed with Mann–Whitney, Wilcoxon and Friedman tests, as appropriate, using Statistica 10 software (StatSoft, Uppsala, Sweden).

## Results

**Figure 2:**
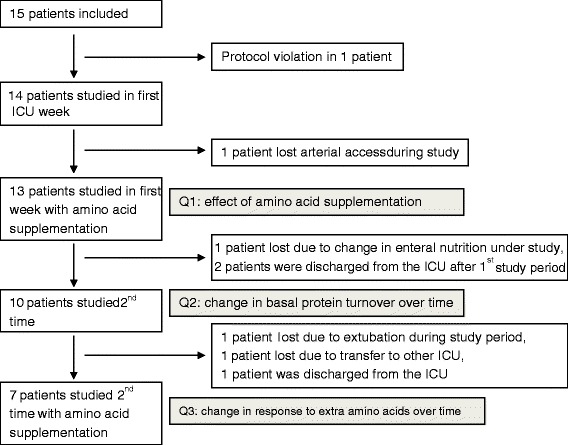
**CONSORT diagram of the number of patients included into the study.** Because of the pragmatic nature of the study, the cohorts of patients studied to answer the three research questions were not identical. ICU, Intensive care unit.

Fifteen critically ill patients were included in the study (Figure 2). Data from 13 patients were used to address the primary question. Of these, ten patients were studied again at baseline to address the second question. Ultimately, seven patients were studied twice, both at baseline and during amino acid supplementation, to address the third question. The characteristics of the patients in the three cohorts are given in Table 1. The three cohorts were comparable with regard to age, sex, weight, diagnosis, Simplified Acute Physiology Score III and Sequential Organ Failure Assessment score. Of the patients included to address question 1, 12 of 13 received insulin and 5 of 13 received noradrenaline during the measurements. Of the patients included to address question 2, ten of ten received insulin on first day and nine of ten on the second day, and five of ten received noradrenaline during the first day and three of ten on second day. Of the patients included to address question 3, seven of seven received insulin during the first day and seven of seven the second day, and five of seven received noradrenaline on day 1 and two of seven on day 2.

The amounts of energy and protein given are shown in Table 2. Because nutrition was given according to clinical routines at the discretion of the attending intensivist, a large variation in the amount of energy and protein given to the patients was observed. Energy and protein intake increased during the intervention period, owing to the amino acid supplementation. However, both energy and protein intake during the baseline periods on the 2 study days were similar.

**Table 2:** **Nutrition**

	**Total energy intake**	**Total protein intake**
	**(kcal/kg/day)**	**(g/kg/day)**
Q1 effect AA (n =13)		
First basal	17 (0 to 26)	0.7 (0.0 to 1.0)
First AA	21 (4 to 30)	1.6 (1.0 to 2.0)
*P*-value	0.001	0.001
Q2 time effect basal (n =10)		
First basal	16 (0 to 21)	0.6 (0.0 to 1.0)
Second basal	18 (0 to 36)	0.7 (0.0 to 1.4)
*P*-value	0.09	0.11
Change from first to second	8 (−21 to 23)	0.3 (−1.0 to 0.9)
Q3 time effect AA (n =7)		
First basal	14 (0 to 20)	0.5 (0.0 to 0.8)
First AA	17 (4 to 24)	1.5 (1.0 to 1.7)
*P*-value	0.017	0.017
Second basal	20 (10 to 36)	0.8 (0.4 to 1.4)
Second AA	24 (14 to 40)	1.7 (1.4 to 2.3)
*P*-value	0.017	0.017

The data reflect the amount of energy and protein given to the three different patients cohorts during the basal and amino acid supplementation (AA) measurements to answer the three research question (Q1 to Q3). Question 1: Do extra parenteral amino acids during the first week in the intensive care unit (ICU) modulate whole body protein kinetics? Question 2: Do baseline protein kinetics change during early ICU treatment? Question 3: Are the effects of extra parenteral amino acids maintained after a few days? *P*-values indicate statistically significant difference from basal (Wilcoxon signed-rank test).

**Figure 3:**
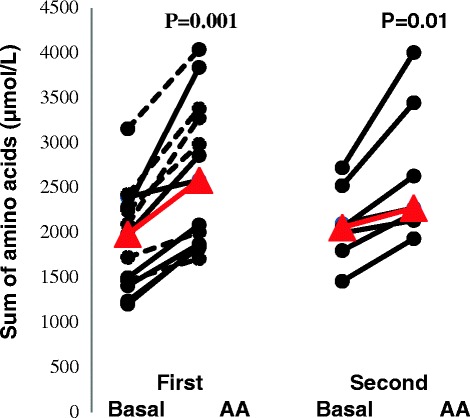
**Sum of all amino acids in plasma of critically ill patients during baseline and parenteral amino acid supplementation (AA) on 2 study days.** On the first study day, 13 patients were studied, and on the second study day, 7 of these patients were still being treated in the intensive care unit and were studied again. The seven patients studied twice are indicated by continuous lines, and the other five studied on day 1 only are indicated by dashed lines. Individual values are shown, with the median in red. The sum of amino acids consists of glutamate, serine, glutamine, histidine, glycine, threonine, arginine, alanine, tyrosine, valine, methionine, tryptophan, phenylalanine, isoleucine, leucine and lysine.

Most amino acids given during amino acid supplementation increased in plasma concentration after 3 hours (see Additional file 2). The sum of total amino acid concentration increased from 2.0 (1.2 to 3.2) mM to 2.6 (1.7 to 4.0) mM on study day 1 (*P* =0.001) and from 2.0 (1.5 to 2.7) mM to 2.3 (1.9 to 4.0) mM on study day 2 (*P* =0.01) (Figure 3).

**Figure 4:**
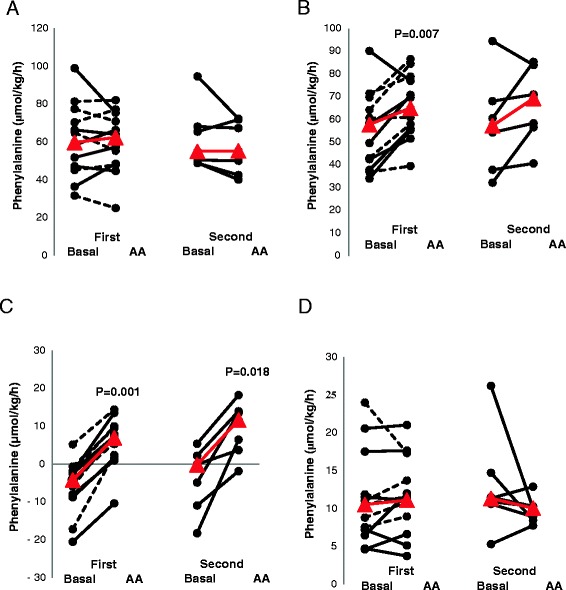
**Whole-body protein breakdown (A), protein synthesis (B), protein balance (C) and phenylalanine oxidation (D) in critically ill patients during baseline and parenteral amino acid supplementation (AA) on 2 study days.** On the first study day, 13 patients were studied, and on the second study day, 7 of these patients were still being treated in the intensive care unit and were studied again. The seven patients studied twice are indicated by continuous lines, and the other five studied on day 1 only are indicated by dashed lines. Individual values are shown, with the median in red.

The results of the protein turnover measurements for the three cohorts are summarized in Table 3. The results for protein synthesis, protein breakdown, protein balance and oxidation (estimated by phenylalanine hydroxylation) for questions 1 and 3 are shown in Figure 4. For the 13 patients assessed on study day 1, protein synthesis and protein balance increased during amino acid supplementation, whereas no changes in protein breakdown or oxidation rates were observed. Splanchnic extraction decreased. For the ten patients studied again at baseline, 2 to 4 days later no changes were observed as compared with the initial baseline measurements. For the seven patients studied at baseline and during amino acid supplementation on both study days, no significant differences in the response to the amino acid supplementation were observed (Table 3). The protein balance increased on both days with amino acid supplementation (Figure 4). The protein synthesis rate increased, which reached statistical significance during the first measurement (from 58 to 65 μmol/kg/hr phenylalanine) (n =13; *P* =0.007), but not during the second measurement (from 58 to 69 μmol/kg/hr phenylalanine) (n =7; *P* =0.09). Protein oxidation rates, estimated based on the phenylalanine hydroxylation rate, were not changed on both days during the amino acid supplementation.

**Table 3:** **Whole-body protein kinetics**

		**Breakdown**	**Synthesis**	**Balance**	**Splanchnic extraction**	**Oxidation**
Q1 effect AA (n =13)						
	First basal	60 (32 to 99)	58 (34 to 90)	−4 (−21 to 5)	0.4 (0.2 to 0.6)	11 (5 to 24)
	First AA	62 (25 to 82)	65 (40 to 87)	7 (−10 to 14)	0.2 (−0.1 to 0.9)	11 (4 to 21)
	*P*-value	0.92	0.007	0.001	0.037	0.47
Q2 time effect basal (n =10)						
	First basal	59 (32 to 99)	54 (34 to 90)	−5 (−21 to 5)	0.4 (0.2 to 0.6)	9 (5 to 21)
	Second basal	60 (35 to 95)	59 (29 to 95)	−5 (−18 to 5)	0.3 (−0.1 to 0.9)	11 (5 to 26)
	*P*-value	0.17	0.45	0.80	0.31	0.20
Q3 time effect AA (n =7)						
	Change from first basal to AA	3 (−24 to 12)	12 (−13 to 21)	10 (9 to 16)	−0.1 (−0.4 to 0.2)	0 (−2 to 6)
	Change from second basal to AA	−1 (−22 to 6)	4 (−11 to 25)	12 (4 to 25)	0 (−0.1 to 0.5)	−1 (−16 to 3)
	*P*-value	0.40	0.50	0.50	0.14	0.40

The data represent whole-body protein turnover rates of the three patient cohorts to answer the three research questions (Q1 to Q3), expressed as micromolar phenylalanine per kilogram of body weight per hour, except for the splanchnic extraction, which is shown as a fraction. Question 1: Do extra parenteral amino acids (AA) during the first week in the intensive care unit (ICU) modulate whole-body protein kinetics? Question 2: Do baseline protein kinetics change during early ICU treatment? Question 3: Are the effects of extra parenteral amino acids maintained after a few days. *P*-values indicate statistically significant difference for question 1 between amino acid supplementation (AA) and basal; for question 2, between basal measurements on the 2 study days; and for question 3, between the change from basal to supplementation on study days 1 and 2, respectively (Wilcoxon signed-rank test).

**Figure 5:**
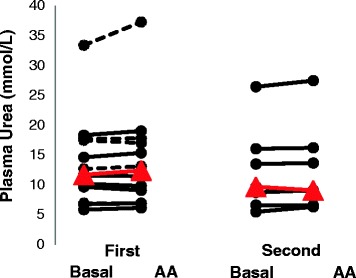
**Plasma urea concentrations in critically ill patients during baseline and parenteral amino acid supplementation (AA) on 2 study days.** On the first study day, 13 patients were studied, and on the second study day, 7 of these patients were still being treated in the intensive care unit and were studied again. The seven patients studied twice are indicated by continuous lines, and the other five studied on day 1 only are indicated by dashed lines. Individual values are given, with the median in red.

Plasma urea levels did not change from baseline during amino acid supplementation (Figure 5).

**Figure 6:**
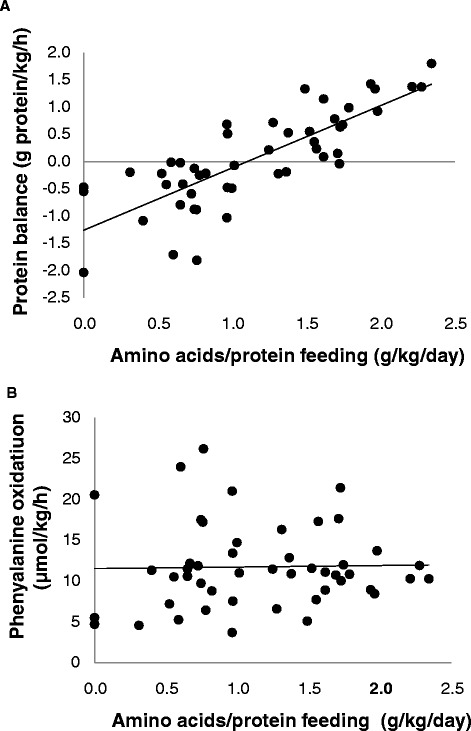
**Statistical correlations between total amino acid and/or protein feeding and whole protein balance (A) and protein oxidation rate (B) in 13 critically ill patients during baseline and parenteral amino acid supplementation (AA) on 2 study days.** On the first study day, 13 patients were studied, and on the second study day, 7 of these patients were still being treated in the intensive care unit and were studied again. All measurements performed for all patients are included. The protein balance is presented in grams of protein per kilogram of body weight per day. This was calculated assuming that the phenylalanine content of the human whole-body protein pool is 4% [12]. A positive correlation with *r* =0.80 (*P* <0.0001) was observed between the amino acid and/or protein feeding and whole-body protein balance. No correlation with phenylalanine oxidation was observed (*r* =0.02; *P* =0.88).

When the actual amino acid and/or protein intake for all the patients at all time points was compared with the protein balance, a positive correlation was observed (*r* =0.80; *P* <0.0001) (Figure 6). No correlation with protein oxidation rates was observed (*r* =0.02; *P* =0.88) (Figure 6). For these comparisons, the protein balance and protein oxidation rates are presented in grams of protein per kilogram of body weight per day. The calculations were performed assuming that the phenylalanine content of the human whole-body protein pool was 4% [12].

## Discussion

The main result of the present study is that, during the first week of ICU treatment, extra amino acids could be used in critically ill patients during a short period of parenteral supplementation to improve whole-body protein balance. When this intervention was repeated 2 to 4 days later, the response was similar. Whole-body protein turnover did not appear to change during the initial week following ICU admission. The improvement in protein balance due to the extra parenteral amino acids was attributable mainly to an increased protein synthesis rate, which attained statistical significance during first study day, but not during the second study day, on which fewer patients were included. In addition, no increase in phenylalanine hydroxylation rate was observed during amino acid supplementation. These results, together with the unchanged plasma urea levels, imply that none of the supplemental amino acids given were oxidized. In a pilot study, we also show that the enteral protein kinetics could be assessed with the addition of a free amino acid tracer to the enteral nutrition, as long as the patients were fed continuously and a 4-hour run-in period was taken into account. We also show, in the pilot study, that enterally given free labeled phenylalanine, instead of an intrinsically labeled protein, could be used in continuously fed critically ill patients to estimate splanchnic extraction for whole-body protein kinetics. This result from the pilot study also shows that these critically ill patients were able to fully digest the protein given to them, in this case casein.

It is well known that many critically ill patients are severely catabolic and lose mainly muscle protein. The most obvious way to prevent or counteract this loss is by protein feeding. However, the recommendations for the amount of protein to be given are based on weak evidence [1]. In addition, the preferred route of administration (parenteral or enteral) remains uncertain. There is also evidence that overfeeding, or even any feeding, during the initial week of an ICU stay might extend ICU stay [13]. It is well established that parenteral amino acid supplementation increases protein balance in healthy humans, with most studies showing that this is attributable to both an increase in protein synthesis rate and a decrease in breakdown rate [14]. Our results suggest that the effect in critically ill patients is attributable mainly to increases in protein synthesis rates. However, these increases could also be a result of the choice of the labeled amino acid because different amino acids tracers can show a similar response on protein balance, but by different mechanisms [9]. Nonetheless, previous work in healthy humans using the same amount of amino acid supplementation as we used in the present study also showed an increase in protein synthesis rates only [15]. Our patients, however, were older than most of the healthy volunteers in these previous studies. However, also in older adults (mean age, 73±3 years), a parenteral amino acid infusion is able to increase whole-body protein balance to the same extend as in younger healthy volunteers [16]. However, for enteral supplementation, this might be different because of increased splanchnic extraction in the older adults [17].

Most studies in which researchers have investigated the effects of parenteral amino acids in critically ill patients and healthy volunteers have been focused on the effect over a few hours only. In the present study, the anabolic effect was observed after 3 hours, but whether these effects are sustained over more prolonged periods was not studied. In preterm infants, an anabolic effect of short amino acid supplementation was observed, but it did not persist after 24 hours [18]. However, in a recent study on the effect of high (2.8 g/kg/day) parenteral amino acid supplementation versus a normal dose (1.5 g/kg/day) in critically ill adolescents, researchers showed that, even after 24 hours of supplementation, a more positive whole-body protein balance could be achieved with the higher dose [19]. However, both these studies included critically ill patients of much younger age than the patient cohort in our present study.

The present study was designed as a pragmatic study, implying that no interference with treatment, including nutritional support, was made. The only intervention was that intravenous glutamine supplementation was stopped several hours before the study because the amino acid supplementation we used also contained glutamine. This pragmatic approach was associated with dropout of patients during the ongoing study and a large variation in the amount of nutrition given. A rather wide range—between 0 and 1.4 g/kg/day—of protein and/or amino acid supply was already observed at the baseline measurements. With the additional parenteral amino acid supplementation, the range for all the measurements increased to between 0 and 2.3 g/kg/day equivalent. When the amount of the actual ongoing protein and/or amino acid feeding was correlated to whole-body protein balance, a positive correlation (*r* =0.80; *P* <0.0001) was observed (Figure 6). No significant correlation was observed between amino acid and/or protein feeding and phenylalanine hydroxylation rates representing protein oxidation rates (Figure 6). These results imply that, during the initial 10 days following ICU admission, additional proteins and/or amino acids could be used in critically ill patients for building protein, irrespective of background nutritional support, and that the supplemental amino acids provided were not oxidized.

The increases in whole-body protein balance and protein synthesis by the extra parenteral amino acid administration in the present study do not reveal where these effects are located. On the basis of the whole-body measurements, it is not clear which proteins were synthesized or in which tissues. In healthy humans, extra parenteral amino acids have been shown to increase muscle protein synthesis [14,15], which also is the preferred target in critically ill patients if the goal is to preserve muscle mass and function. However, several studies have shown that the loss of muscle protein in the critically ill is mainly the result of an increased protein breakdown rate and not of a decreased synthesis rate [20-22]. Thus, the extra parenteral amino acids may also be used to synthesize other proteins in different tissues. Specific studies measuring protein turnover of specific proteins or tissues are necessary to elucidate this.

The strengths of our study are that we used a pragmatic approach with minimal alterations in standard care, making the results applicable to real-life settings, and that we used a balanced amino acid supplement that included all amino acids. Many commercial parenteral amino acid solutions lack both glutamine and tyrosine because of instability and low solubility. The solution we used contains both these amino acids in the form of dipeptides (glycyl-glutamine and glycyl-tyrosine). The limitations of the study are that we studied the effect during only a relatively short period of amino acid and/or protein supplementation and that we assessed only whole-body protein turnover and not the specific proteins or tissues.

## Conclusions

Supplemental parenteral amino acids can be used in critically ill patients to stimulate whole-body protein accretion during the initial week following ICU admission. The anabolic properties of such supplemental amino acid administration can be effectively repeated within merely a few days, still within the first week of ICU treatment. Longer-term intervention studies are needed to assess whether the anabolic properties of supplemental amino acid provision can be sustained.

## Key messages

- Supplemental parenteral amino acids can be used in critically ill patients for body protein accretion during the first week of ICU treatment.
- Extra parenteral amino acids given to critically ill patients over the course of 3 hours are not oxidized.
- Whole-body protein turnover does not change in critically ill patients during the initial week following ICU admission.

## Additional files

Additional file 1:
**Data from the pilot study.** Plasma enrichment and rate of appearance measured with ^13^C-phenylalanine intrinsically labeled casein protein and with the same amount of free [^2^H_5_]phenylalanine given enterally together with maltodextrin in six critically ill patients for 6 hours (individual data). Additional file 2:
**Plasma amino acid concentrations of all amino acids.** Plasma amino acid concentrations (μmol/L) in critically ill patients at baseline (basal) and after 3 hours of extra parenteral amino acids equivalent to 1 g/kg/day (Glavamin; Fresenius-Kabi).

## Abbreviations

AA: Period during which the patients received extra intravenous amino acids as Glavamin
ICU: Intensive care unit
MPE: Molar percent excess
SAPS III: Simplified Acute Physiology Score III
SOFA: Sequential Organ Failure Assessment
