# Supplementary material for: Short-term amino acid infusion improves protein balance in critically ill patients
Source: Crit Care. 2015 Mar 12;19(1):106. doi: 10.1186/s13054-015-0844-6 (PMC4403712; doi:10.1186/s13054-015-0844-6)
Supplement: Additional file 1: — Data from the pilot study. Plasma enrichment and rate of appearance measured with 13C-phenylalanine intrinsically labeled casein protein and with the same amount of free [2H5]phenylalanine given enterally together with maltodextrin in six critically ill patients for 6 hours (individual data). [file 13054_2015_844_MOESM1_ESM.pdf]

Supplementary file 1:

Plasma enrichment (A) and rate of appearance (B) measured with ^{13}C-phenylalanine intrinsically labeled casein protein (35.2 MPE) and with the same amount of free $^{2}H_5$ phenylalanine (99.7 MPE) given enterally together with maltodextrin in six critically ill patients for 6 hours (individual data). The rate of appearance is calculated from the two different tracers by dividing the enteral infusion rate by the plasma enrichment of each tracer at each time point. In one patient none of the tracers escaped the splanchnic tissues, so for this patient (patient 5) no rate of appearance could be calculated. After about 4 hours the rate of appearance was identical for both tracers.

A

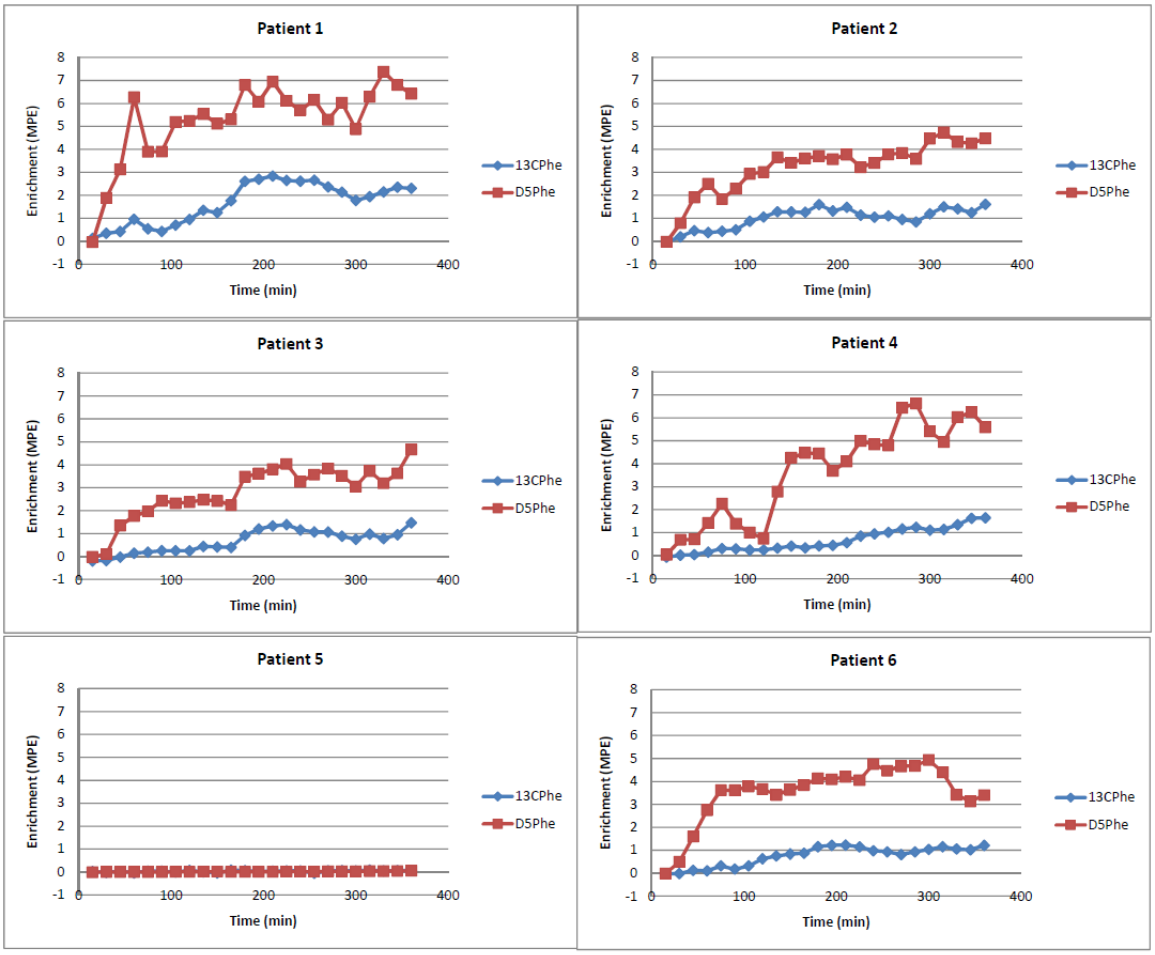

B

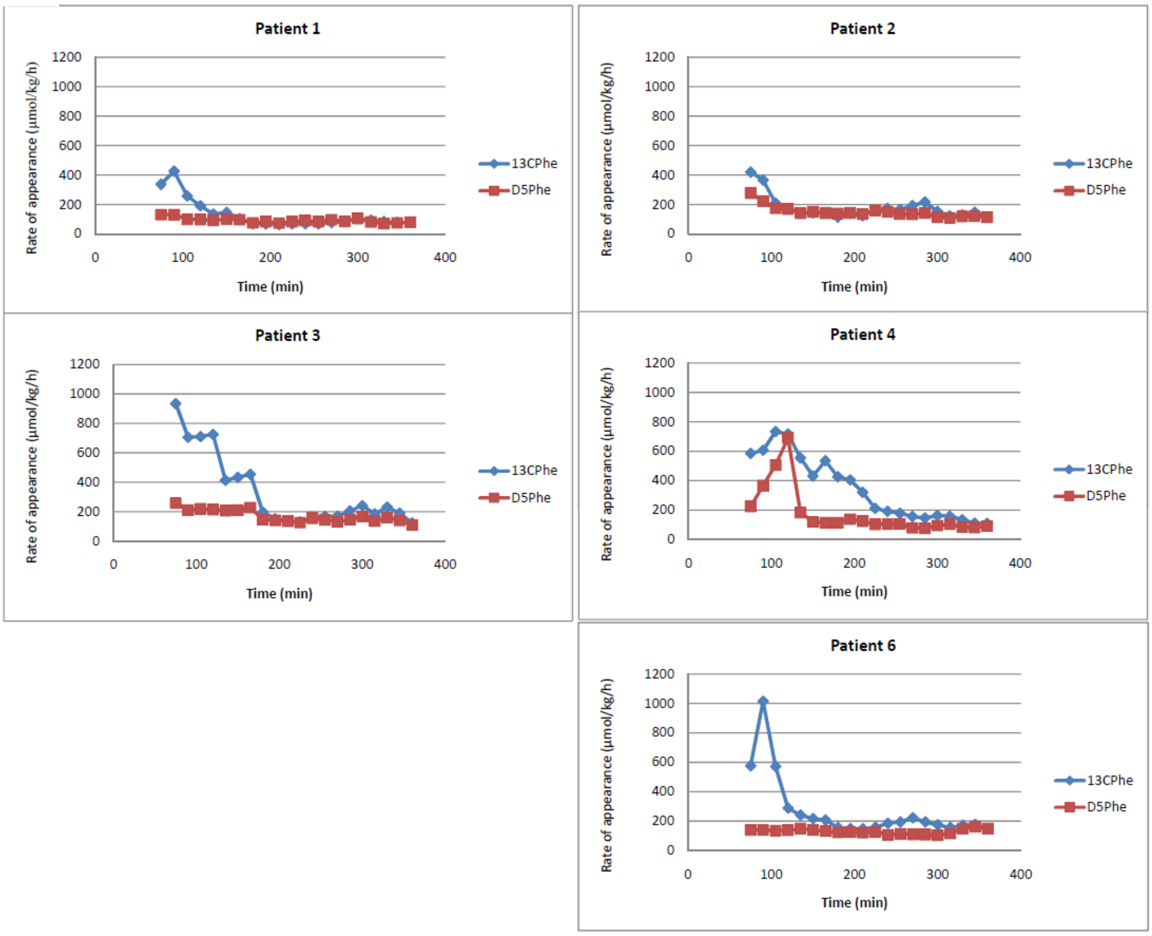
Patient 1
Patient 2
Patient 3
Patient 4
Patient 6
Rate of appearance (µmol/kg/h)
Time (min)
0
100
200
300
400
0
200
400
600
800
1000
1200
13CPhe
D5Phe
