# Supplementary material for: Short-term amino acid infusion improves protein balance in critically ill patients
Source: Crit Care. 2015 Mar 12;19(1):106. doi: 10.1186/s13054-015-0844-6 (PMC4403712; doi:10.1186/s13054-015-0844-6)
Supplement: Additional file 2: — Plasma amino acid concentrations of all amino acids. Plasma amino acid concentrations (μmol/L) in critically ill patients at baseline (basal) and after 3 hours of extra parenteral amino acids equivalent to 1 g/kg/day (Glavamin; Fresenius-Kabi). [file 13054_2015_844_MOESM2_ESM.pdf]

Supplementary file 2:

Plasma amino acid concentrations (µmol/L) in critically ill patients at baseline (basal) and after 3 hours extra parenteral amino acids (Glavamin, Fresenius-Kabi; equivalent of 1 g/kg/day). On the first study day, 13 patients were studied and on the second study day 7 of these were still treated in the ICU and studied again.

	First Study day				Second Study day				Statistics (P= ;Paired t-test)	
	Basal		AA		Basal		AA		First	Second
	Mean	SD	Mean	SD	Mean	SD	Mean	SD	Basal vers. AA	Basal vers. AA
**Glutamate**	**65.0**	15.8	**71.2**	11.7	**69.3**	15.5	**67.1**	21.3	*0.4906*	*0.7874*
**Serine**	**44.9**	12.5	**49.8**	13.4	**47.6**	9.5	**47.9**	11.5	*0.2349*	*0.4743*
**Aspragine**	**68.1**	14.7	**88.7**	21.0	**73.1**	9.6	**91.8**	22.5	*0.0081*	*0.2271*
**Glutamine**	**442.7**	45.2	**532.6**	88.7	**499.5**	115.0	**563.5**	165.2	*0.0226*	*0.6739*
**Histidine**	**60.6**	12.5	**87.2**	18.0	**57.8**	4.4	**72.1**	9.7	*0.0171*	*0.2179*
**Glycine**	**154.8**	29.0	**189.7**	40.6	**179.9**	61.9	**228.5**	105.4	*0.0053*	*0.2326*
**Threonine**	**87.4**	22.4	**158.7**	28.4	**92.1**	18.0	**164.8**	43.9	*0.0014*	*0.0649*
**3-Methylhistidine**	**7.0**	3.6	**6.9**	4.8	**6.7**	3.8	**7.2**	4.3	*0.9376*	*0.0503*
**Citrulline**	**18.1**	1.5	**21.5**	3.7	**20.0**	5.1	**23.4**	10.7	*0.0316*	*0.5195*
**Arginine**	**62.6**	18.0	**102.8**	30.1	**75.6**	14.1	**119.4**	33.8	*0.0030*	*0.1057*
**Alanine**	**230.4**	64.7	**301.0**	104.7	**222.0**	47.9	**295.5**	90.3	*0.0239*	*0.1963*
**Taurine**	**58.8**	19.6	**58.6**	31.7	**80.1**	20.2	**59.8**	19.9	*0.9807*	*0.3799*
**Tyrosine**	**85.3**	13.4	**103.2**	13.9	**77.7**	3.4	**92.2**	7.5	*0.0098*	*0.1611*
**Valine**	**218.0**	33.0	**293.3**	51.4	**252.7**	27.9	**331.2**	64.8	*0.0078*	*0.0935*
**Methionine**	**26.5**	11.0	**46.4**	16.7	**27.9**	7.4	**49.0**	10.7	*0.0042*	*0.0279*
**Tryptophane**	**28.8**	7.8	**38.2**	9.4	**33.4**	9.8	**39.1**	15.4	*0.0174*	*0.3529*
**Phenylalanine**	**149.1**	66.3	**189.6**	64.7	**150.6**	22.7	**171.8**	5.4	*0.0078*	*0.1893*
**Isoleucine**	**50.2**	8.0	**77.4**	15.5	**62.6**	12.9	**88.2**	16.5	*0.0085*	*0.0402*
**Ornithine**	**65.6**	36.2	**92.7**	49.4	**56.7**	6.4	**67.6**	6.8	*0.0198*	*0.0903*
**Leucince**	**103.7**	17.5	**138.4**	24.3	**118.2**	7.6	**150.1**	17.1	*0.0150*	*0.0884*
**Lysine**	**157.9**	38.5	**213.5**	53.1	**172.9**	31.3	**230.1**	54.5	*0.0072*	*0.1746*
**BCAA**	**360.4**	96.2	**526.9**	172.2	**383.2**	74.8	**524.2**	91.8	*0.0000*	*0.0024*
**SUM**	**1913.0**	533.5	**2621.0**	785.0	**2141.5**	392.9	**2665.0**	714.3	*0.0000*	*0.0058*
